# What is the impact of blood pressure on neurological symptoms and the risk of ESKD in primary and secondary thrombotic microangiopathies based on clinical presentation: a retrospective study

**DOI:** 10.1186/s12882-022-02672-3

**Published:** 2022-01-20

**Authors:** Jean-Michel Halimi, Benjamin Thoreau, Florent von Tokarski, Adeline Bauvois, Juliette Gueguen, Nicolas Goin, Christelle Barbet, Sylvie Cloarec, Elodie Mérieau, Sébastien Lachot, Denis Garot, Adrien Lemaignen, Emmanuel Gyan, Franck Perrotin, Claire Pouplard, François Maillot, Philippe Gatault, Bénédicte Sautenet, Emmanuel Rusch, Véronique Frémeaux-Bacchi, Cécile Vigneau, Guillaume Bayer, Fadi Fakhouri

**Affiliations:** 1 Service de Néphrologie-HTA, Dialyses, Transplantation Rénale, Hôpital Bretonneau et hôpital Clôcheville, CHU Tours, 2 Bd Tonnellé, 37044 Tours Cedex, France; 2 EA4245, François-Rabelais University, Tours, France; 3 Service d’Hématologie Biologique, Hôpital Bretonneau, CHU Tours, Tours, France; 4 Service de Médecine Intensive Réanimation, Hôpital Bretonneau, CHU Tours, Tours, France; 5 Service de Maladies Infectieuses, Hôpital Bretonneau, CHU Tours, Tours, France; 6 Service d’Hématologie et Thérapie Cellulaire, Hôpital Bretonneau, CHU Tours; ERL CNRS 7001, Université de Tours, Tours, France; 7 Service de Gynécologie Obstétrique B, Maternité Olympe de Gouges, Hôpital Bretonneau, CHU Tours, Inserm U1253 « Imaging and Brain », François-Rabelais University, Tours, France; 8 Laboratoire d’Hématologie-Hémostase, Hôpital Trousseau, CHU Tours, Tours, France; 9 EA7501, François-Rabelais University, Tours, France; 10 Service de Médecine interne, Hôpital Bretonneau, CHU Tours, Tours, France; 11 Inserm U1246, Hôpital Bretonneau, CHU Tours, Tours, France; 12 Laboratoire de Santé Publique, Hôpital Bretonneau, CHU Tours, Tours, France; 13 Laboratoire d’Immunologie, Hôpital Européen Georges Pompidou, Paris, France; 14 CHU Pontchaillou, service de néphrologie, 35033 Rennes, Université Rennes 1, Inserm IRSET, UMR 1085, 35033 Rennes, France; 15 Service of nephrology, department of medicine, CHUV and Université de Lausanne, Lausanne, Switzerland

**Keywords:** Blood pressure, hypertension, epidemiology, thrombotic microangiopathy, ESKD, neurological symptoms, posterior reversible encephalopathy syndrome

## Abstract

**Background:**

The impact of blood pressure on neurological symptoms and risk of end-stage kidney disease (ESKD) is unknown in primary and secondary thrombotic microangiopathies (TMAs).

**Methods:**

We measured baseline systolic (SBP) and diastolic (DBP) BP in consecutive 563 patients with adjudicated primary and secondary TMAs, and assessed its association with the risk of ESKD.

**Results:**

Normal BP, grade 1, 2 and 3 hypertension were present in 243 (43.1%), 132 (23.4%), 101 (17.9%) and 88 (15.6%), respectively.

Significant BP differences were noted in relation to the cause of TMA: highest BP values were found in patients with atypical hemolytic-uremic syndrome (aHUS), pregnancy, transplantation and auto-immune-related TMAs. Normal BP or grade 1 hypertension was found in 17/18 (94.4%) patients with thrombotic thrombocytopenic patients (only 1/18 (5.6%) had a SBP value>150 mmHg). In contrast, BP values could not differentiate isolated “essential” malignant hypertension (MH) from MH associated with aHUS (isolated MH (n=15): BP (median (IQR)): 220 (182-249)/132 (101-150) mmHg; MH with aHUS (n=5): BP: 223 (196-245)/131 (111-144) mmHg).

The risk of vigilance disturbances (6.9%, 15.0%, 25.0%, respectively), epileptic seizures (1.5%, 4.0%, 12.5%, respectively) and posterior reversible encephalopathy syndrome (0.76%, 2.97%, 6.82%, respectively) increased with increasing baseline BP values from grade 1 to grade 3 hypertension.

ESKD occurred in 35/563 (6.2%) patients (1.23%, 2.27%, 11.9% and 19.3% of patients with normal BP, grade 1, 2 and 3 hypertension, respectively). As compared to patients with normal BP (<120/139 mmHg), grade 1, grade 2 and grade 3 hypertension were associated with a greater risk of ESKD in univariate (OR: 1.91 [0.83-4.40], 13.2 [3.56-48.9] and 34.8 [9.31-130], respectively) and multivariate (OR: 0.89 [0.30-2.69], 7.00 [1.57-31.3] and 19.7 [4.53-85.2], respectively) analyses. The association between BP and the risk of ESRD was unchanged after adjustment on eculizumab use (OR: 3.46 [1.41-8.49], 17.7 [4.44-70.0] and 70.6 [8.61-579], respectively). Patients with MH, regardless of its cause, had a greater risk of ESKD (OR: 26.4 [10.0-69.8] vs other patients).

**Conclusions:**

Baseline BP differs in primary and secondary TMAs. High BP reduces the neurological tolerance of TMAs and is a powerful independent risk factor of ESKD, even after adjustment on TMA’s cause.

## Background

Thrombotic microangiopathy (TMA) is a heterogenous group of diseases characterized by thrombocytopenia and mechanical hemolytic anemia with schistocytosis and elevated lactate dehydrogenase (LDH) [1]. They represent a diagnosis and therapeutic challenge for clinicians, and are associated with a poor renal outcome in the most severe cases [1, 2]

Little is known regarding the relationship between blood pressure (BP) and TMAs. This issue has been overlooked as BP values are not even reported in large-scale TMA studies [3–8]. Nonetheless, some data suggest that the interactions between BP and TMA are important. Firstly, endothelial injury plays a pivotal role in both TMA and severe hypertension [1, 9]. Secondly, TMA may induce hypertension, via mainly renal ischemia, and conversely severe hypertension may lead to TMA [10]. Thirdly, severe hypertension may interfere with the pathogenic mechanism of various TMA, and it has been linked to an activation of the complement alternative pathway [11, 12] and to a reduction in ADAMTS13 activity [13]. However to date, whether baseline BP differs according to the cause of TMA, and whether BP has a distinct impact on outcomes in TMAs are unknown. Any information regarding the epidemiological value of BP in TMAs could shed some light on the pathophysiology of acute hypertension and essential malignant hypertension [12, 13].

The aim of the present retrospective study was to assess the association between baseline BP and causes of TMA, and to evaluate the impact of between baseline BP on renal survival in a large cohort of consecutive patients with a wide range of adjudicated TMA.

## Methods

### Selection of patients

Patients with suspected TMA who were admitted to the Tours university hospital (France) between January 1^st^, 2009 and December 31^st^, 2016 were included. As previously described [14], patients were identified using 2 modes of detection: the presence of schistocytosis in the laboratory results and/or the presence of specific keywords in hospitalization discharge summaries (HDS). All patients’ records were reviewed individually (manually) using all available data by 4 physicians (AB, GB, FVT, BT), including medical reports and electronic databases and diagnosis of TMA was confirmed or ruled out. TMA was suspected based on the presence of at least 3 of the following criteria: hemoglobin <12 g/dL, increased LDH, low haptoglobin and schistocytosis ≥0.5% associated with thrombocytopenia (platelets count <150 G/L) [1, 2]. Cases were adjudicated by three physicians familiar with the management of TMA and practicing in Competence Centers [14].

The first step of the adjudication was to rule in or rule out the diagnosis of TMA. Most causes of TMA are thrombocytopenic thrombotic purpura (TTP, due to severely reduced activity of ADAMTS13 (A Disintegrin And Metalloproteinase with ThromboSpondin-1 motifs, 13th member)), atypical hemolytic and uremic syndrome (aHUS, mostly due alternate pathway complement defects), shiga toxin associated TMA (STEC-HUS), TMA associated with pregnancy (usually due to pre-eclampsia, HELLP (hemolysis and elevated liver enzymes and low platelet count), post-partum hemorrhage (PPH)), STEC-unrelated infections, transplantation, malignancies, auto-immune diseases and medications. The second step was to identify the cause of TMA using a strict hierarchical process: first, presence of ADAMTS13 activity ≤10% for the diagnosis of TTP. In the absence of TTP, diagnosis of HUS-STEC was considered in the presence of shiga toxin-producing E. Coli using stool cultures and/or PCR. Then, pregnancy-related TMA was suspected in patients with HELLP, pre-eclampsia or severe delivery bleeding. The same hierarchical process was applied for other causes of TMAs (TMAs associated with specific drugs, transplantations, STEC-unrelated infections, cancers, auto-immune disease and severe/malignant hypertension (hypertensive retinopathy and usually diastolic arterial pressure>120 mmHg). In patients with TMA and renal failure but none of the above-mentioned TMA causes, aHUS was suspected. Some rare patients had none of the above-mentioned diagnoses: we described their clinical and biochemical presentation (“other TMA” group).

### Baseline BP and renal outcome

BP at baseline was collected and categorized as normotension (systolic/diastolic blood pressure (SBP/DPB)<140/90 mmHg), grade 1 hypertension (140-159/90-99 mmHg), grade 2 hypertension (160-179/100-109 mmHg) and grade 3 hypertension (≥180/110 mmHg). In some patients, antihypertensive medications were started before hospital admission; BP values before the start of antihypertensive medications were recorded when available; if not, baseline BP at admission was noted. Malignant hypertension was clinically defined as a severe diastolic BP (DBP) (>120 mmHg) with neurological-associated symptoms or papilledema on funduscopic examination [15], regardless of the underlying cause of TMA (some patients had isolated essential malignant hypertension whereas others had malignant hypertension associated with other causes of TMAs) [14].

Acute kidney injury (AKI) was defined using the KDIGO criteria [16]. Only serum creatinine criteria were used to diagnose and stage AKI (urinary output criteria were omitted). Dialysis was recorded during hospitalization. Renal recovery at 90 days was noted to differentiate acute dialysis (dialysis duration ≤90 days) from end-stage renal disease (ESRD) (dialysis duration >90 days) [16].

### Statistical analyses

Quantitative data are presented as median and interquartile range (IQR). Qualitative data are described with percentages. Comparisons were made using Chi square test or Fischer test as appropriate for qualitative data and Wilcoxon test for quantitative data. Univariate and multivariate logistic regressions were performed for the identification of parameters associated with the risk of ESRD. SAS software (version 9.3) was used.

## Results

### Baseline characteristics

We identified 564 patients with TMA during the 2009-2016 period [17]. Baseline blood pressure (BP) value was available in 563/564 (99.8%) patients who are included in the present study. Overall, median age was 37 (IQR: 27-57) and two-third of patients were females. TMA features (thrombocytopenia (92.2%), anemia (96.5%), low haptoglobin levels (90.4%) and schistocytes (79.3%)) were present in most patients (Table 1).

**Table 1:**
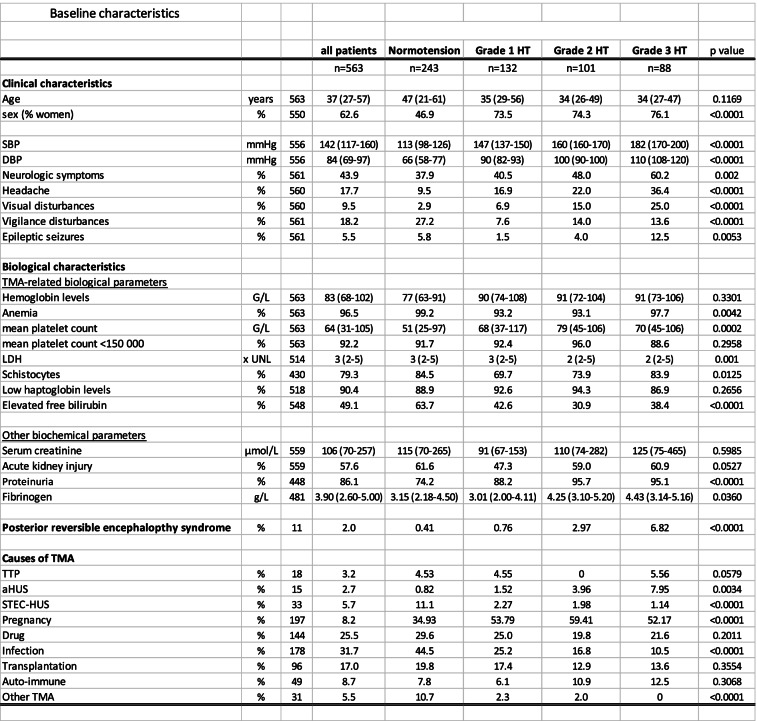
Baseline characteristics

Median SBP/DBP were 142 (117-160)/84 (69-97) mmHg. Normal BP, grade 1, grade 2 and grade 3 hypertension were present in 243 (43.1%), 132 (23.4%), 101 (17.9%) and 88 (15.6%) patients, respectively (Table 1). Although significant differences regarding mean platelet count, LDH, haptoglobin and hemoglobin were noted across BP groups of patients, there was no clear dose-effect association between BP and hematological severity of TMA (Table 1). However, patients with the highest BP values presented more often with AKI, proteinuria, seizures, headache, visual disturbances and posterior reversible encephalopathy syndrome (PRES) (Table 1).

### BP and causes of TMA

Significant differences in BP categories were noted among TMA causes: highest BP values were found in patients with aHUS, pregnancy, transplantation and auto-immune-related TMAs.

In contrast, normal BP or grade 1 hypertension was found in most patients with TTP and infection-related TMAs (Table 1). When patients with TTP or aHUS were considered together, values provided interesting information regarding the cause of TMA: among patients with TTP, 0/18 (0%) had a DBP value>100 mmHg and only 1/18 (5.6%) had a SBP value>150 mmHg (vs 10/15 (66.7%) and 10/15 (66.7%), respectively, in patients with aHUS (both p<0.0001)). Thus, these BP cut-off values were useful to suspect the diagnosis of TTP (negative predictive value for TTP: 100%) or suspect aHUS (positive predictive value for aHUS: 78.0%) in the absence of other obvious TMA causes.

In contrast, BP values could not differentiate malignant essential hypertension from aHUS in some patients. Among the 15 patients with aHUS, 5 had malignant hypertension whereas among the 20 patients with malignant hypertension, 5 patients had aHUS and 15 had malignant essential hypertension: their BP was similar (SBP/DBP: 220 (182-249)/132 (101-150) (n=15) vs 223 (196-245)/131 (111-144) mmHg (n=5)). Among the 15 patients with aHUS, complement studies (including genetics) indicated that 12/15 (80%) patients had complement abnormalities (low serum C3 levels (n=3), low CD46 expression on granulocytes (n=2), factor H variant (n=2), C3 mutation (n=2). Among the 5 patients with aHUS and malignant hypertension, 2 (40%) had a factor H mutation.

### Renal outcome

#### Acute dialysis

**Fig 1:**
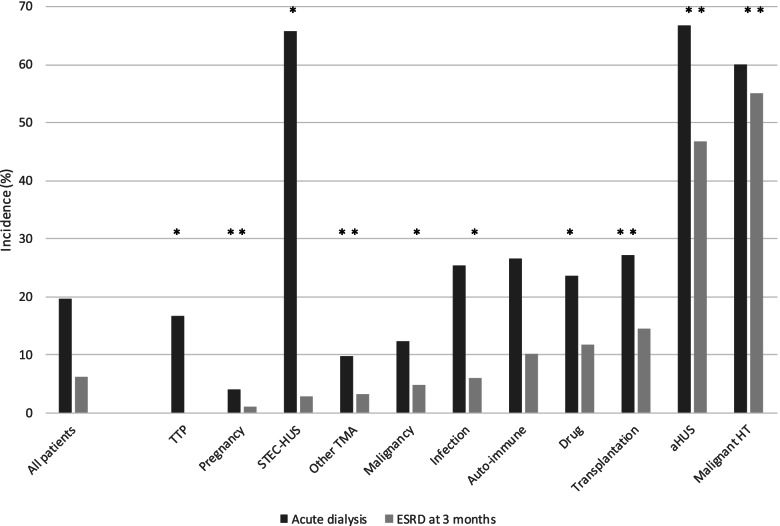
Proportion of acute dialysis during hospitalization and ESRD at 3 months according to the cause of thrombotic microangiopathy

**Fig. 2:**
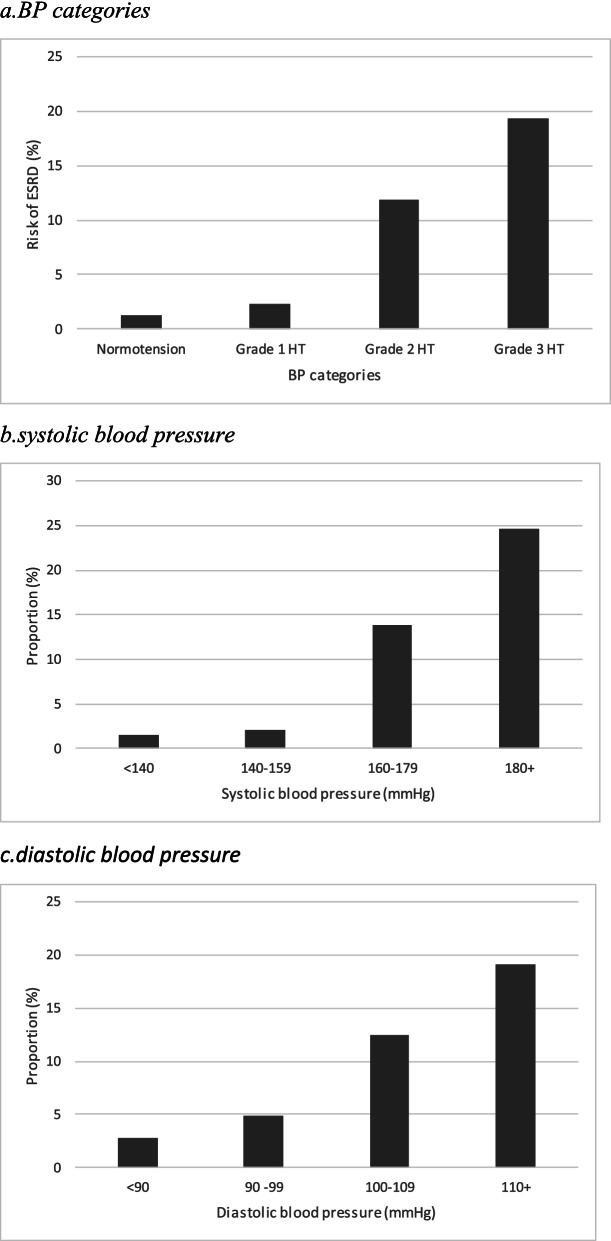
Proportion of ESRD at 3 months in relation to baseline blood pressure. **a**.BP categories. **b**.systolic blood pressure. **c**.diastolic blood pressure

During hospitalization, 111/563 (19.7%) patients needed dialysis and 55/563 (9.8%) patients died (Fig.1). Acute dialysis was more frequent in patients with TMA related to aHUS (66.7%, p<0.0001 vs other patients), STEC-HUS (65.7%, p<0.0001 vs other patients), malignant hypertension (60.0%, p<0.0001 vs other patients), transplantation (27.1%, p=0.0452 vs other patients) and infections (25.5%, p=0.0101 vs other patients), and less frequent in patients with pregnancy-related TMA (4.1%, p<0.0001 vs other patients) (Fig.1). There was a J-curve relationship between BP categories and the proportion of acute dialysis (20.6%, 18.3%, 12.9% and 11.4% for normal BP, grade 1 hypertension, grade 2 hypertension and grade 3 hypertension groups, respectively) (Fig. 2).

#### ESRD at 3 months

At 3 months, ESRD occurred in 35/111 (31.5%) of patients with acute dialysis (35/563 (6.2%) patients), more frequently in patients with TMAs related to drugs (11.8%, p=0.0012 vs other patients), transplantation (14.6%, p=0.0002 vs other patients), aHUS (46.7%, p<0.0001 vs other patients) and malignant hypertension (55.0%, p<0.0001, vs other patients). ESRD was not observed in patients with TTP, and rare in patients with pregnancy-related TMAs (Fig.1).

In multivariate analyses, aHUS (odds ratio (OR): 6.50 [1.71-24.7]), pregnancy (OR: 0.21 [0.05-0.94]), malignant hypertension (OR: 26.4 [10.0-69.8]) and transplantation (OR: 3.63 [1.78-7.44]) were significantly associated with ESRD in multivariate analyses (Table 2).

**Table 2:**
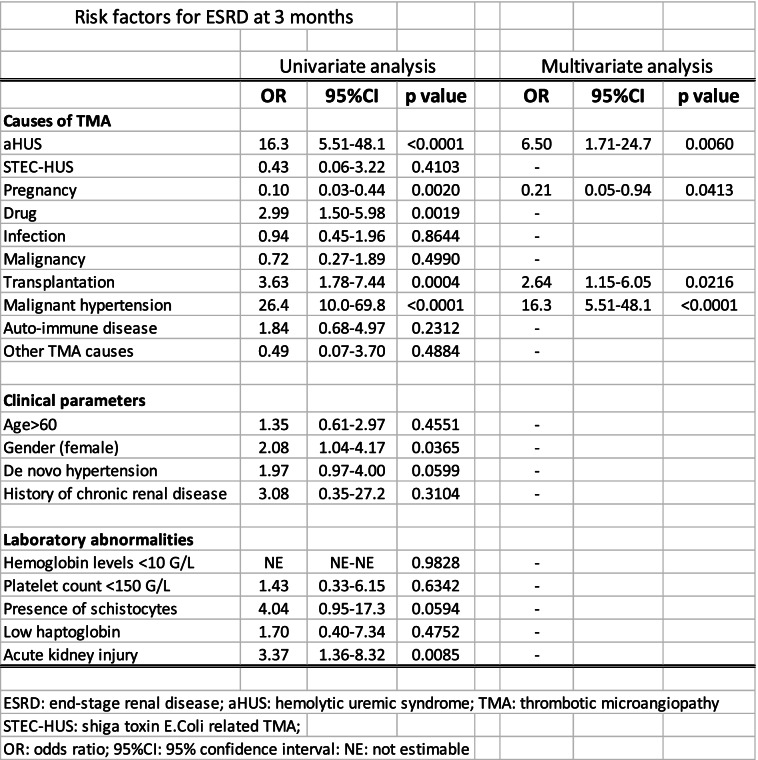
Risk factors for ESRD at 3 months

ESRD occurred in 1.23%, 2.27%, 11.9% and 19.3% of patients with normal BP, grade 1, grade 2 and grade 3 hypertension groups, respectively (Fig.2a); similar dose-effect relationships between BP categories and the proportion of ESRD were found for SBP (1.54%, 2.05%, 13.86%, 24.56%, respectively, p<0.0001) (Fig.2b) and DBP (2.74%, 4.90%, 12.5%, 19.1%, respectively, *p*<0.0001) (Fig.2c).

In univariate analyses, BP was a powerful risk factor for ESRD: there was a dose-response relationship across BP categories and the risk of ESRD (Table 3). These results remained significant in multivariate analyses in all models used (Table 3).

**Table 3:**
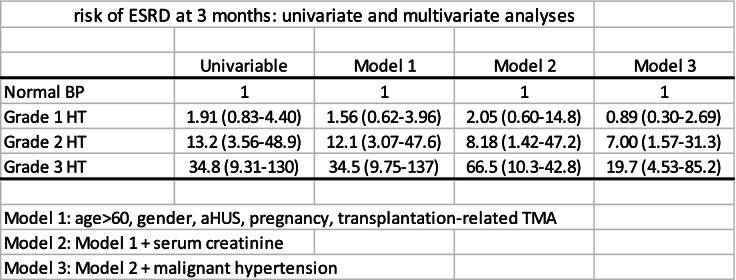
risk of ESRD at 3 months: univariate and multivariate analyses

Interestingly, eculizumab was used in 10/15 (66.7%) of patients with aHUS. Of note, since 2012, only 1 patient with aHUS was not treated with eculizumab: she remained on dialysis (the diagnosis of factor H mutation was made several months after the disease onset). Importantly, aHUS was no longer a risk factor for ESRD when eculizumab and BP categories were entered into the models (OR: 2.80 [0.53-14.8], p=0.2263). Nevertheless, the association between BP and the risk of ESRD was unchanged after adjustment on eculizumab use (vs SBP<120 mmHg: OR (120-139 mmHg): 3.46 [1.41-8.49], p=0.0067); OR (140-179 mmHg): 17.7 [4.44-70.0], p<0.0001; OR (≥180 mmHg: 70.6 [8.61-579], p<0.0001).

## Discussion

We assessed the epidemiological value of BP in a large cohort of patients with various types of TMAs. Files were individually reviewed and therefore identification of included patients was not based on administrative codes but careful analysis of clinical and biological data. All consecutive TMA cases were included, thus reducing selection bias. They were adjudicated by experienced physicians.

Our first finding is that BP significantly differed across distinct causes of TMA. These differences allowed better identification of the causes of TMA. BP value at baseline was a powerful diagnostic tool: among patients with TTP, values of BP >150 mmHg for SBP or 100 mmHg for DBP virtually excluded the diagnosis of TTP (negative predictive value for TTP: 100%). When no other obvious cause was present, these BP cut-off value allowed a strong suspicion of aHUS in most cases (positive predictive value for aHUS: 78.0%). The identification of the cause of TMA is crucial, and any delay negatively affects patient survival [1, 18]. Data on BP values in TMA in the literature are scarce. In 17 patients with TMA, BP values was not reported but the percentage of hypertension appeared similar in patients with secondary TMA and primary STEC-HUS, aHUS and TTP [8]. In another recent report, complement gene variants were detected in 8 patients with severe hypertension and features of TMA [12], underscoring the possibility that malignant hypertension may be a presenting feature of aHUS [12]. Our own data support this view. We believe that BP may be an additional parameter that could help clinicians rapidly distinguish aHUS from TTP in emergency settings [19, 20].

The second finding is that there was a striking dose-response relationship between baseline BP and the risk of ESRD, regardless of the cause of TMA. Interestingly, the BP-related risk of ESRD was not restricted to patients with severe or malignant hypertension, and not even restricted to patients with hypertension as it started at a normal SBP value (120 mmHg). Moreover, BP remained the major parameter associated with the risk of ESRD in multivariate analyses. In 62 patients with TMA, Dierkes et al found that elevated arterial pressure was a risk factor for persistent renal disease [21]. Jammes et al recently indicated that BP was a risk factor for chronic renal disease in patients with aHUS untreated by eculizumab [22]. Our results are important as they apply to various types of TMAs, regardless of their causes.

The nature of the relationship between BP and the pathophysiology of TMA is not clearly understood. Hypertension probably results from severe endothelial damage, a common feature to both TMAs and severe hypertension [19]. However, we did not identify a dose-response relationship between BP levels and the severity of TMA as exemplified by the presence of schistocytes, serum level of haptoglobin, LDH, hemoglobin and platelet counts. In contrast, proteinuria and acute kidney injury were frequent in patients with the highest BP values, suggesting renal severity but not hematological severity of TMA plays a major role in BP levels. TMAs provoke acute reversible renal lesions (i.e. thrombi in arteries, thickening and obliteration of the small artery lumen, fibrinoid necrosis of arterial wall), and these lesions may heal or regress after resolution of TMAs [19]. Our results suggest that either high BP aggravates the renal lesions of TMAs or that high BP is a marker of severe and sometime irreversible renal lesions. In the absence of renal biopsy, it is difficult to analyze these findings.

Interestingly, neurological symptoms such as headaches, visual disturbances, seizures and PRES were frequently observed in patients with severe BP values, regardless of the cause of TMAs. These findings suggest that high BP plays a major role in the neurological tolerance of TMA. They also support the widespread view that BP lowering to normal levels must be achieved in hypertensive patients with TMAs to ensure a more complete recovery of TMA symptoms [23–25]. Alternatively, it is possible that thrombi in the cerebral circulation may lead to sympathetic nerve activation and subsequent increased BP, as it is observed in patients with ischemic stroke [26–28]

Interestingly, in a recent study in 20 patients with malignant hypertension, half of patients had low haptoglobin, and 12 patients had reversible encephalopathy syndrome. Among these 12 patients, 11 patients with reversible encephalopathy syndrome had both cortex and brainstem lesions. In addition, 6/7 patients with headache at presentation had reversible encephalopathy syndrome whereas 6/12 without headache had also reversible encephalopathy syndrome. These lesions disappeared after BP control [29].

Autopsy studies revealed that microthrombi are present in the kidneys of most patients with TTP, typically affecting few segments of the glomeruli, but there is no significant renal infiltration of inflammatory cells in these patients unlike patients with other causes TMA and AKI in whom these lesions are more widespread and more severe [30, 31]). These differences may explain the BP difference between TTP and with other causes of TMA such as aHUS, as less severe renal lesions may results in abnormal BP regulation by the kidneys. Moreover, baseline serum creatinine was lower in patients with TPP (1.3 [1.0-1.7 mg/dl]) than in most patients with other causes of TMA (aHUS : (4.6 [1.7-7.9 mg/dl] ; STEC-HUS : 4.8 [0.8-7.1 mg/dl] ; auto-immune diseases (2.2 [1.1-3.6 mg/dl] ;transplantation (1.9 [1.3-3.6 mg/dl]) in our study. In this view, high BP could reflect widespread parenchymal renal damage, and may not be the direct cause of AKI and subsequent ESKD. It is also important to note that patients with TTP were usually younger (38 [IQR : 31-51] than many other patients in our study (transplantation-related TMA (51 [41-63]), auto-immune diseases (51 [31-65]) which could play a lower risk of increased BP before the onset of TMA in patients with TTP.

The results of the present study are robust but they come from a single institution, and therefore they need to be replicated using a prospective study design. The results of renal biopsies could certainly shed some light on the nature of the association between baseline BP and renal survival, as it was shown that some patients with specific causes of renal diseases (such as IgA nephropathy) can present with high BP and TMA, and that the risk of ESRD was elevated in this population [32]. Kidney biopsy should probably be discussed in most patients with TMAs and initial acute kidney injury, especially in the presence of high BP [19, 31].

## Conclusion

Our data indicate that TMAs, a group of severe hematological diseases, diversely affect the acute regulation of BP, and that BP should be carefully analyzed in patients with TMA. Epidemiological studies focused should report baseline BP values as these values provide valuable information regarding the identification of the cause of TMAs, which in turn can lead to reduction of delays in diagnosis and therapy and improved prognosis [1, 18, 33] [34]. BP value is associated with poor neurological tolerance of TMA, and strongly suggests that strict BP control is warranted in this population. Finally, BP value,-not TMAs’ hematological severity- is an excellent marker of irreversible renal damage associated with TMAs, and is a powerful independent determinant of renal survival in TMAs.

## Data Availability

the datasets generated and/or analyzed during the current study are not publicly available due to the fact was registered as an internal research, not as public research, but are available from the corresponding author on reasonable request”. Database is available for audit.

## Abbreviations

ADAMTS13: A Disintegrin And Metalloproteinase with ThromboSpondin-1 motifs, 13th member
aHUS: Atypical hemolytic and uremic syndrome
AKI: Acute kidney injury
ESRD: End-stage renal disease
HELLP: Hemolysis and elevated liver enzymes and low platelet count
IQR: Interquartile range
MH: Malignant hypertension
PPH: Post-partum hemorrhage
SBP/DPB: Systolic/diastolic blood pressure
STEC-HUS: Shiga toxin associated TMA
TMA: Thrombotic microangiopathy
TTP: Thrombocytopenic thrombotic purpura
